# Influence of scan mode, tilt, and radiation dose on CT radiomic metrics

**DOI:** 10.1002/acm2.70462

**Published:** 2026-01-14

**Authors:** Neha Yadav, Xiaomeng Lei, Steven Y. Cen, Joshua Levy, Kristin Jensen, Bino A. Varghese

**Affiliations:** ^1^ Keck School of Medicine University of Southern California Los Angeles California USA; ^2^ The Phantom Laboratory Greenwich New York USA; ^3^ Department of Physics and Computational Radiology Oslo University Hospital Oslo Norway

**Keywords:** image acquisition, phantom study, quality control, radiomic analysis

## Abstract

**Background:**

Radiomic features derived from computed tomography (CT) are highly susceptible to variations in acquisition parameters, which can introduce confounding effects in multicenter research and reduce diagnostic accuracy. While the effects of parameters such as scanning mode and dose have been studied, the impact of gantry tilt—despite its routine clinical use—remains underexplored in radiomics literature.

**Purpose:**

To systematically evaluate how scan mode (axial vs. helical), gantry tilt (0° vs. 5°), and radiation dose affect CT‐based radiomic metrics using an anthropomorphic liver phantom containing six 3D‐printed texture inserts, with special emphasis on the novel inclusion of tilt.

**Methods:**

Twelve unique image acquisition configurations were scanned on a GE Revolution Apex CT scanner, with each configuration repeated once. Manual segmentation of volumes of interest (VOIs) was performed, and 93 radiomic features spanning six texture families were extracted using PyRadiomics. First‐order dispersion metrics (standard deviation, interquartile range, and coefficient of variation) were analyzed alongside higher‐order features via regression with heatmap visualization, and repeatable, robust, and calibratable features were identified.

**Results:**

Helical scans without tilt generally exhibited lower first‐order dispersion than axial scans. Introducing a 5° tilt reduced dispersion in axial scans but had inconsistent effects in helical scans, with no coherent trend observed. Radiation dose demonstrated an expected inverse relationship with dispersion metrics. Intraclass correlation coefficient (ICC) analysis revealed that 34% of radiomic metrics exhibited good or excellent repeatability across all trials (ICC ≥ 0.6), but only 13% demonstrated good or excellent robustness, highlighting the sensitivity of radiomic metrics to scanning conditions. Regression analysis yielded 31 metrics (33%) that can be calibrated using their significant linear relationships with the parameters varied in this study, thereby allowing researchers to correct for variations in acquisition settings.

**Conclusions:**

These findings underscore the importance of accounting for acquisition variability—including less frequently examined parameters such as tilt—when designing radiomic studies, selecting robust features, and interpreting results in clinical and multicenter studies. This approach helps distinguish meaningful biological variation from imaging artifacts, thereby improving the reliability of radiomic analysis in personalized medicine.

## 1 INTRODUCTION

Radiomics is a rapidly growing field in oncology that enables the high‐throughput extraction of quantitative features from routine medical images.^1^, ^2^ Its central premise is that images harbor latent information on tissue heterogeneity that may not be perceptible through conventional visual assessment.^3^ By characterizing patterns, textures, and intensity distributions, radiomics enhances the interpretive value of standard scans—allowing for whole tumor assessment without additional imaging or radiation exposure, as radiomic analysis can be performed retrospectively.^4^ This approach enhances diagnostic precision, supports clinical decision‐making, and is particularly valuable in addressing the increasing diagnostic workload faced by radiologists, offering a potential decision‐support tool that complements expert interpretation.^5^, ^6^

Despite its promise, clinical translation of radiomics is hindered by the lack of quality control standards.^3^ Radiomic analyses often rely on retrospective data, leaving image acquisition parameters beyond the investigator's control. In multicenter studies, variability in scanner hardware, acquisition techniques, and reconstruction settings can significantly influence feature values, potentially obscuring true biological differences and inflating false‐positive rates.^7^ To achieve robust and reproducible results, it is critical to quantify how acquisition variability—such as differences in scanning mode, radiation dose, or tilt—affects radiomic analysis.^8^

Previous work has shown that several acquisition factors can markedly alter first‐order texture measures. For example, slice thickness and radiation dose have been reported to inversely correlate with dispersion metrics such as standard deviation (SD), interquartile range (IQR), and coefficient of variation (CV).^9^, ^10^ Reconstruction method was also found to impact feature stability, whereas tube voltage was shown to exert minimal influence on first‐order metrics.^10^, ^11^ While the findings discussed in the literature shed light on some influential parameters, the effects of other parameters, including tilt, remain underexplored. Clinically, gantry tilt is frequently used to reduce direct radiation exposure to radiosensitive organs and to avoid artifact‐generating regions of the body.^12^ For example, in head CT, gantry tilt is used to reduce radiation exposure to the eyes and to enable imaging of the temporal bone while excluding metal artifacts due to teeth fillings.^12^, ^13^ However, tilt can cause shifts in the isocenter and deviations in the lateral direction as the gantry angle increases.^14^ It can also affect spatial resolution and lead to wider section sensitivity profiles in multi‐planar reconstruction images, potentially impacting radiomic feature values extracted from such images.^15^ Therefore, it is critical to understand the impact of varying this parameter on radiomic metrics.

The present study extends prior work by examining the influence of scan mode, tilt, and radiation dose on radiomic features. In clinical CT practice, these three acquisition parameters are often adjusted simultaneously to optimize coverage, accommodate patient anatomy, and reduce exposure. Evaluating their combined effects provides a more realistic assessment of radiomic feature stability across routinely varied acquisition settings. By systematically isolating these factors in a controlled phantom setting, we are able to identify which features remain stable or require calibration, thereby guiding the selection of robust features for clinical and research applications.

## 2 METHODS

Axial and helical CT scans of an anthropomorphic liver phantom were acquired on a 16 cm detector GE Revolution Apex CT scanner (GE Healthcare, Waukesha, Wisconsin), with variations across dose and tilt (Table 1).

**TABLE 1:** Image acquisition parameters.

Imaging parameter	Settings
Scanning mode	Helical
	Axial
Tilt	0° (No tilt)
	5° Tilt (reconstructed)
Radiation dose	100% (13.86 mGy)
	40% Reduction
	60% Reduction

### 2.1 Phantom description

**FIGURE 1:**
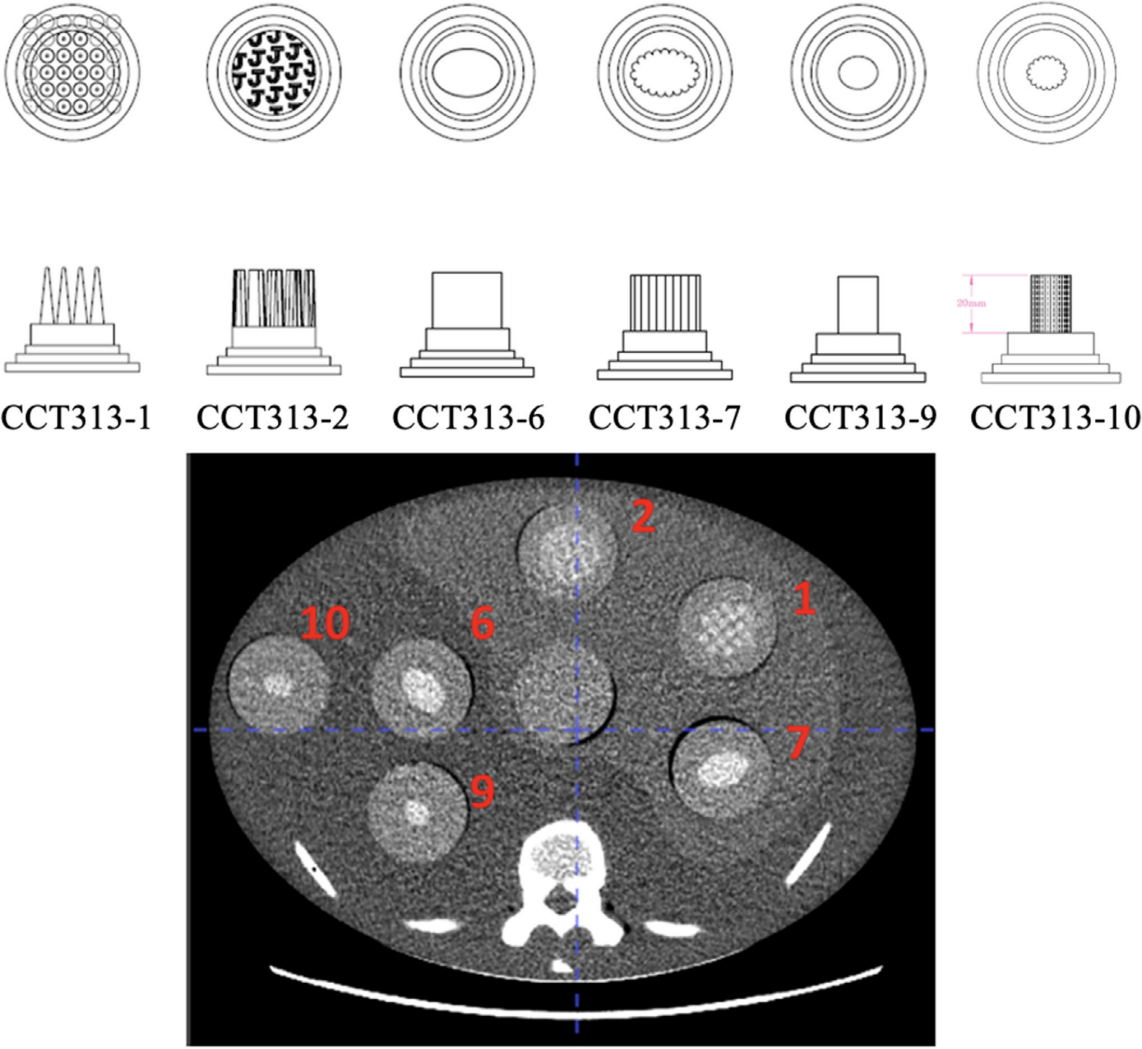
CT Texture Analysis (CTTA) phantom with six 3D‐printed texture inserts. The top row illustrates the pattern embedded within each insert; the middle row illustrates the shape of the insert; and the bottom image depicts the location of each insert in the phantom image.

The CT Texture Analysis (CTTA) phantom (The Phantom Laboratory, Salem, NY) used in this study was modeled after an anthropomorphic liver phantom (The Phantom Laboratory, Salem, NY), which is a simplified human torso featuring an oval cross‐section of 25 cm × 35 cm and a length of 15 cm. Constructed from RANDO tissue‐simulating material, the phantom incorporated differentiated components representing the liver (higher‐density tissue material), vertebrae, and ribs (simulated bone materials), accurately mimicking anatomical densities. The phantom contained seven cylindrical cavities (5 cm diameter), six of which were populated with texture inserts (CCT313_1,2,6,7,9,10) (The Phantom Laboratory, Salem, NY) (Figure 1). These inserts, a subset selected from a prior extensive evaluation of 18 inserts, were selected for their stability in radiomic feature extraction, exhibiting less than 10% variation compared to standard references, including the liver phantom, in vivo liver scans, and water phantoms.

Each insert contained 3D‐printed internal cone‐shaped features fabricated using acrylic‐based materials, embedded in a clear urethane matrix. This design introduced heterogeneity in the *z*‐dimension, a novel attribute not present in existing phantoms. The conical features simulate complex tissue textures relevant for CTTA studies, while the diverse material properties enhance contrast resolution, facilitating reliable assessment of CT texture analysis metrics under controlled experimental conditions.

### 2.2 Imaging parameters

Axial and helical CT scans of the CTTA phantom were acquired on a 16 cm‐wide detector GE Revolution Apex CT scanner. Twelve distinct volumetric CT images were obtained using combinations of three controlled variables: scanning mode (helical, axial); radiation dose (reference dose of 13.86 mGy (CTDIvol), 40% dose reduction, and 60% dose reduction); and tilt (no tilt, 5° tilt). Scans were acquired using automatic dose modulation, varying noise index to obtain the correct reduced doses. The 5° tilting effect was introduced during the image reconstruction phase via image reslicing, using the GE AW Server 3.2 Ext. 4.9 software.^16^ To assess repeatability, each imaging configuration was repeated once, yielding a total of 24 images.

The slice thickness was fixed at 3 mm for all acquisitions to eliminate variability due to partial volume effects. Image reconstruction was performed using standard Filtered Back Projection (FBP) to maintain a consistent reconstruction kernel and noise texture. Tube voltage was held constant at 120 kVp to avoid spectral differences in tissue attenuation. A pitch of 0.5 was used for all helical scans acquired. Table 1 summarizes the acquisition parameters varied in this study.

### 2.3 Volume of interest (VOI) segmentation

**FIGURE 2:**
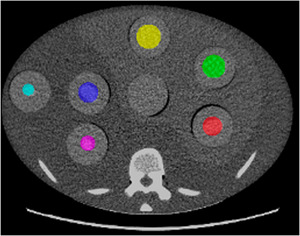
Manual segmentation performed on CT Texture Analysis (CTTA) phantom (volumes of interest (VOIs) indicated by the colored regions). Each texture insert is shown in a distinct color to facilitate visual differentiation.

Manual segmentation was performed to delineate six circular VOIs contoured around the 3D‐printed texture inserts embedded in the phantom (Figure 2). Segmentation was performed using an open‐source software package, CapTk (version‐1.8.1), with care taken to ensure the VOIs encompassed only the intended insert material and excluded surrounding phantom structures.^17^ Each VOI was defined in all contiguous slices containing the insert, thereby generating volumetric segmentations rather than single‐slice regions of interest.

### 2.4 Radiomic feature extraction

Radiomic features were extracted from each segmented VOI using the open‐source PyRadiomics platform (version 3.1.0).^18^ In total, 93 features were computed per VOI, spanning six feature classes as defined by the Image Biomarker Standardisation Initiative (IBSI): First Order, Gray Level Co‐occurrence Matrix (GLCM), Gray Level Run Length Matrix (GLRLM), Gray Level Size Zone Matrix (GLSZM), Gray Level Dependence Matrix (GLDM), and Neighborhood Gray Tone Difference Matrix (NGTDM).^8^

All feature extractions were performed on non‐discretized Hounsfield Unit (HU) data, using PyRadiomics. In alignment with prior studies, a fixed bin width (FBW) discretization scheme with a bin width of 25 HU, the default in Pyradiomics, was used across all VOIs.^19^, ^20^ A fixed bin width of 25 HU provides an optimal balance between preserving texture detail and minimizing noise. Narrow bins (e.g., 1–10 HU) increase sensitivity to minor intensity variations and amplify noise, whereas wide bins (e.g., ≥50 HU) cause volume averaging and structural blurring that obscure texture detail.^21^ Results from the literature (Larue et al., 2017 and Li et al., 2020) support that a bin width of around 25 HU yields the highest proportion of reproducible features across scanners and settings, while reproducibility drops markedly at 1 HU and image fidelity degrades at 50 HU.^21^, ^22^ The FBW approach was selected because CT intensities are quantitative in HU. Maintaining a constant HU interval per gray level preserves the physical meaning of attenuation values and allows for reliable comparison across acquisitions and phantom inserts. The FBW approach was selected over the fixed bin number (FBN) approach because the latter rescales each ROI's dynamic range, reducing reproducibility and physical interpretability. Apart from this, no other image preprocessing tasks were performed before feature extraction.

### 2.5 Statistical analysis

Our analysis was divided into three parts: first‐order metric analysis, focusing on simplistic dispersion measures such as standard deviation (SD), interquartile range (IQR), and coefficient of variation (CV); repeatability and robustness analysis; and regression analysis, assessing all extracted radiomic features for significant linear associations with image acquisition parameters.

**FIGURE 3:**
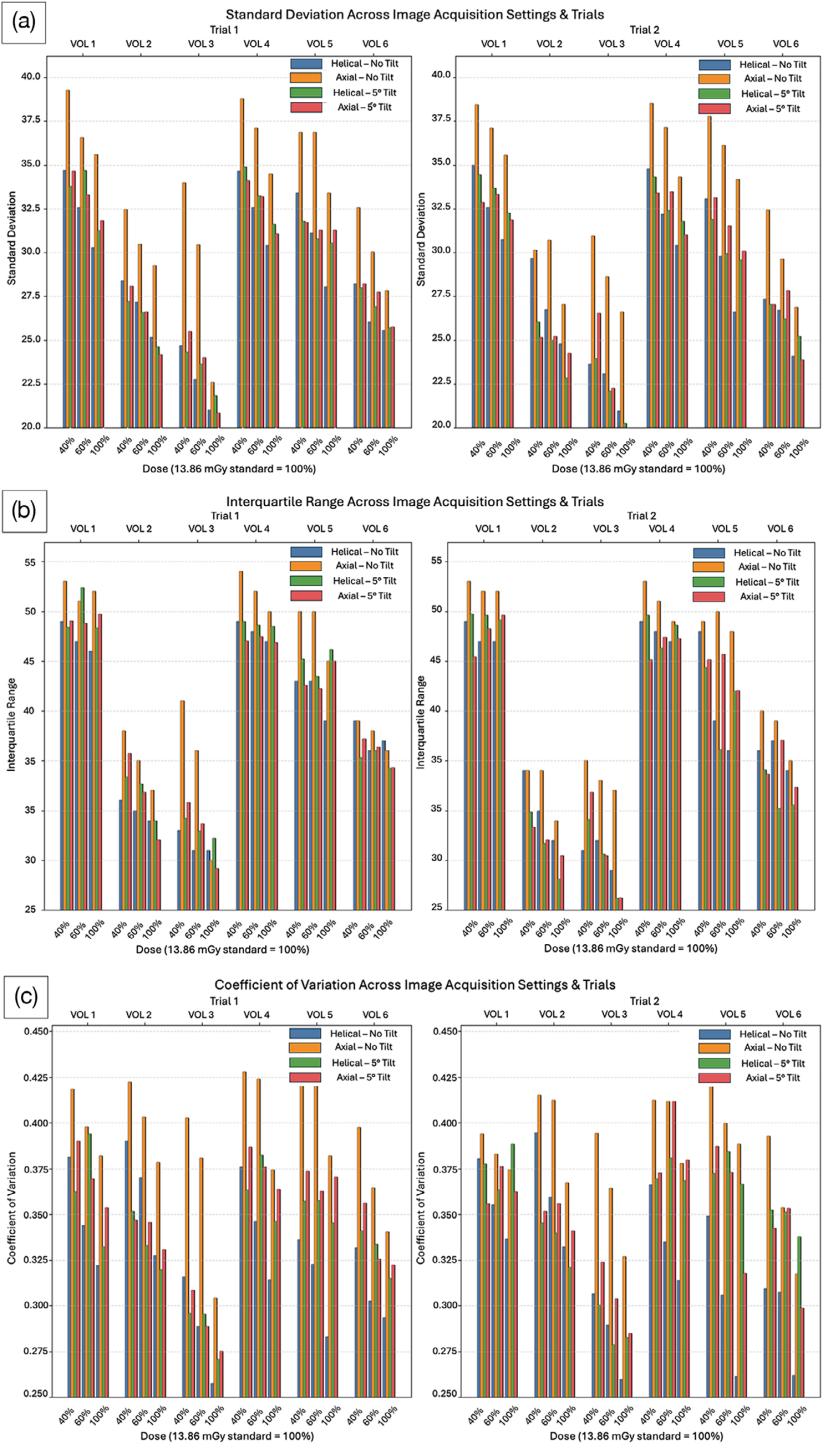
Standard deviation (SD) (a), interquartile range (IQR) (b), and coefficient of variation (CV) (c) across image acquisition settings and trials for all six volumes of interest. Each cluster of bar graphs is grouped by volumes of interest (VOI) (upper *x*‐axis) and dose (bottom *x*‐axis). Within each cluster, the respective first‐order metric is plotted for each combination of scanning mode and tilt (four combinations total). Left: Trial 1; Right: Trial 2 (containing the repeatability data).

SD, IQR, and CV were computed for each VOI across all acquisitions. The changes between acquisition subgroups: 1) axial–no tilt versus axial–tilt; 2) axial–no tilt versus helical–no tilt; 3) helical–no tilt versus helical–tilt; and 4) axial–tilt versus helical–tilt were quantitatively assessed and plotted in a side‐by‐side bar chart (Figure 3). The effect of dose reduction on SD, IQR, and CV was also evaluated.

Both repeatability and robustness were assessed by intraclass correlation coefficient 2‐way mixed, with absolute agreement (ICC(3,1)). Confidence intervals were not calculated for ICC; rather, the nominal value of ICC was used. Repeatability was assessed by comparing ICC(3,1) across trials for each metric, while robustness was measured by computing ICC(3,1) of each of the radiomic metrics across 24 different settings, using a combination of the variables listed in Table 1. The ICC values were categorized based on established thresholds from the literature (Cicchetti, 1994): excellent defined as ICC ≥ 0.75; good defined as 0.60 ≤ ICC < 0.75; fair defined as 0.40 ≤ ICC < 0.60; and poor defined as ICC < 0.40.^23^, ^24^

**FIGURE 4:**
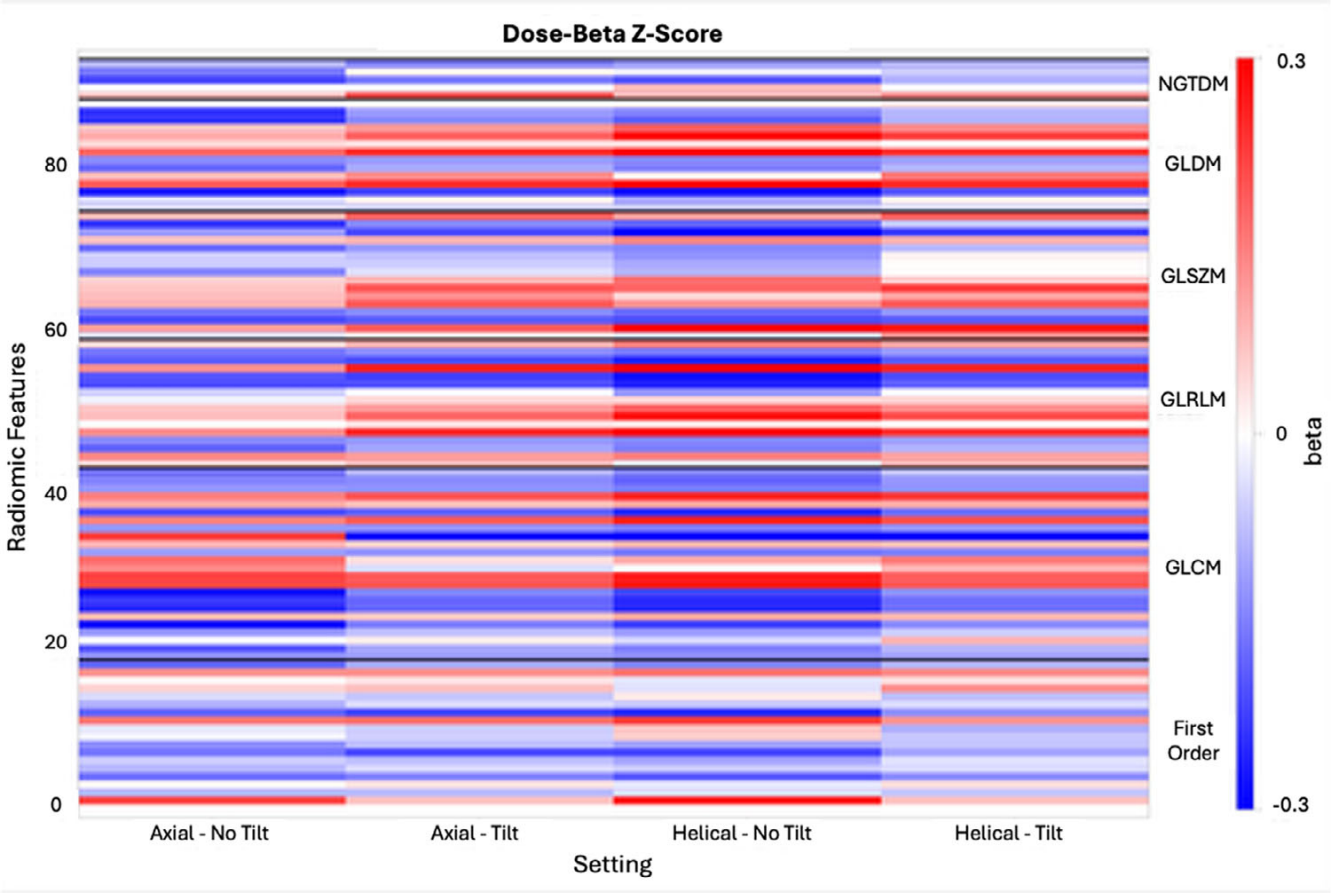
Heatmap of radiomic features across different scanning modes, radiation doses, and image tilting. The *x*‐axis represents the setting of experiments and the *y*‐axis represents the ID of each radiomic feature bundled by texture family (right legend). Each row of the heatmap corresponds to 1 of the 93 radiomic features, grouped by feature family (First Order, GLCM, GLRLM, GLSZM, GLDM, and NGTDM). Each column represents a specific imaging configuration (combinations of scan mode, tilt, and dose, although dose is not explicitly shown in the axis legend). The heatmap uses color intensity to indicate the magnitude and direction of the linear association between each radiomic feature and the acquisition parameter being assessed. Warm colors (red) denote a positive linear correlation, where the feature value increases with the parameter, while cool colors (blue) indicate a negative correlation. Features appearing as white or neutral‐colored bands do not exhibit a strong linear association with the parameters varied in this study.

Additionally, the linear association between image acquisition parameters and all 93 radiomic features was assessed using the Gaussian regression model. For each acquisition parameter of interest (scan mode, dose, or tilt), models were stratified by all possible combinations of the other two parameters to account for interaction effects. For example, the association between dose and radiomic feature values was computed separately for each of the four scanning mode/tilt combinations (two scanning modes * two tilt conditions). The regression analysis included 12 unique image acquisitions and 6 VOIs, for a total of 72 independent observations per model. Repeated acquisitions were excluded from the regression set to prevent artificial inflation of statistical significance due to non‐independence among samples. Regression coefficients (β) were summarized in the form of a heatmap, with the β‐value color‐coded by magnitude and sign (Figure 4). Features were grouped by category (First Order, GLCM, GLRLM, GLSZM, GLDM, and NGTDM) to enable interpretation of parameter sensitivities across texture families. The Benjamini–Hochberg procedure was used to control false discovery rate. Corrections were stratified by scan mode/dose/tilt subsets. Features exhibiting a statistically significant linear association (adjusted *p*‐value ≤ 0.05) with the parameters varied in this study were identified as being calibratable, which means the heterogeneity can be corrected for using a linear model (Appendix A).

### 2.6 Intra‐ and inter‐observer variability

The use of manual segmentation, though common in radiomics studies, introduces the risk of intra‐ and inter‐observer variability. To assess intra‐observer variability in this study, we randomly selected 1 out of the 24 image acquisitions for the analysis (axial–tilt, 40% dose, Trial 1). VOI segmentation was performed twice on that volume, across all slices, by a single operator. Then, ICC(3,1) of both segmentation trials was computed to assess intra‐observer variability. The same volume was subsequently segmented by a different operator, and the ICC(3,1) values across operators were compared to assess inter‐observer variability. A single representative acquisition was sufficient for this phantom study, as segmentation variability depends primarily on operator consistency rather than acquisition parameters. Considering the anatomy and texture complexity remain the same across scans, a single acquisition captures the observer‐dependent variability while avoiding redundancy across similar scans.

## 3 RESULTS

### 3.1 First‐order metrics

SD, IQR, and CV were plotted across all 12 image acquisition configurations (and their respective repeat scans) and all 6 VOIs (Figure 3). Analysis of SD and CV revealed that axial–no tilt consistently produced higher dispersion values compared to helical–no tilt (Figure 3a,c). IQR, however, did not exhibit a consistent trend between these two modes, likely due to its robustness to outliers. Extreme voxel intensities are excluded from IQR calculations, whereas SD and CV include them, potentially amplifying scan mode‐dependent differences.

Introduction of a 5° tilt in axial scans resulted in decreased SD, IQR, and CV relative to the non‐tilt axial scans, indicating that tilting reduces dispersion through partial averaging or blurring effects (Figure 3b). In contrast, the effect of tilt in helical scans was inconsistent: first‐order metrics did not systematically increase or decrease with tilt, suggesting that additional factors—such as volumetric overlap and interpolation artifacts—may affect the measured signal.

No clear pattern was observed when comparing axial–tilt with helical–tilt. This finding likely reflects compounded effects of scanning mode and tilt: helical acquisitions with tilt involve spiral x‐ray paths with overlapping projections, whereas axial–tilt maintains sequential slice acquisition with minimal interpolation, resulting in inherent differences in VOI signal composition.

Radiation dose exhibited the expected inverse relationship with first‐order dispersion: higher doses corresponded to reduced SD, IQR, and CV, consistent with reduced image noise at elevated exposure levels.^9^, ^10^

### 3.2 Repeatability and robustness analysis

**FIGURE 5:**
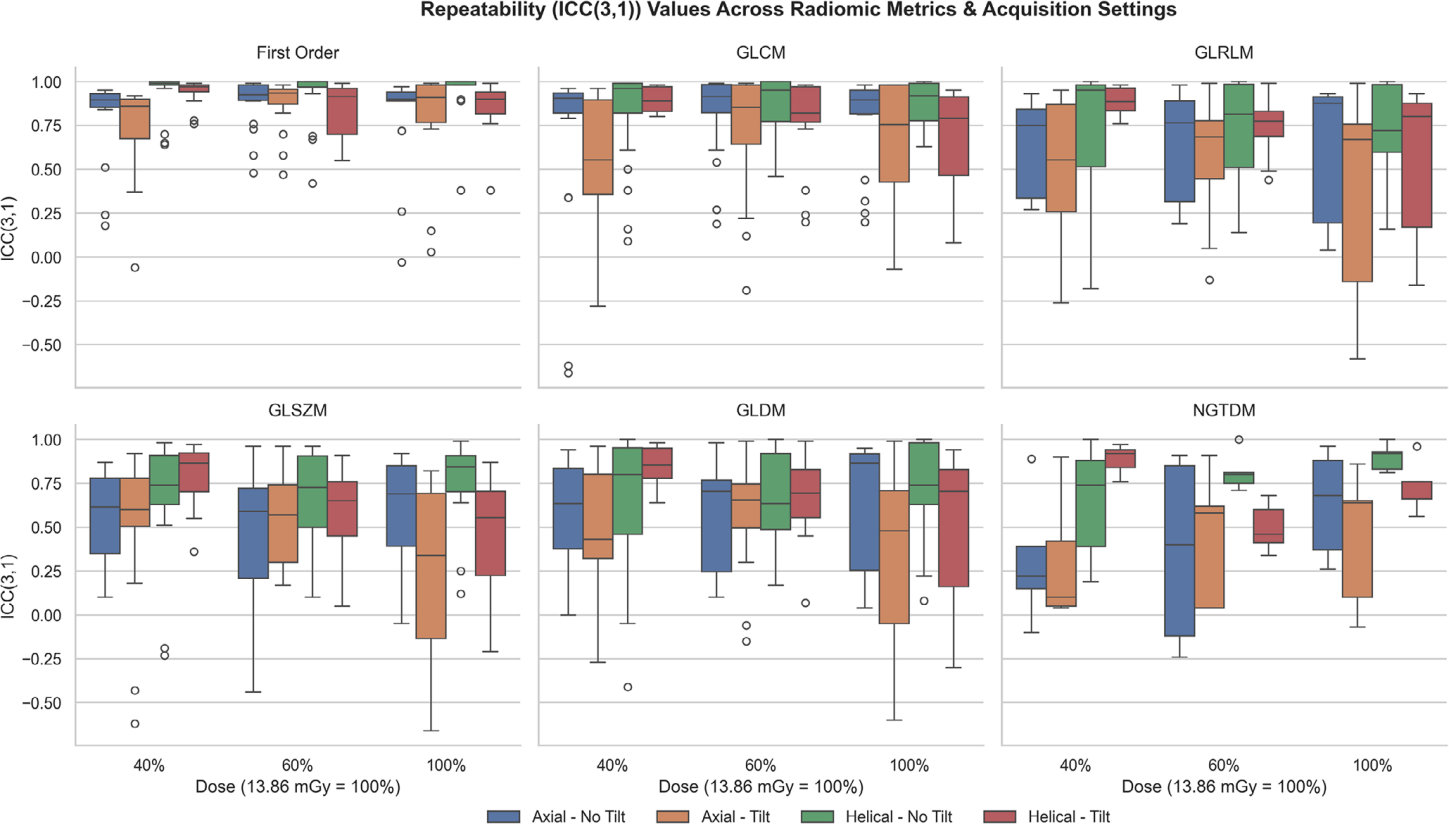
Repeatability (ICC(3,1)) values for each radiomic metric, grouped by feature family, across all image acquisition settings.

Repeatability of the extracted radiomic metrics was assessed using ICC(3,1) to compare features extracted from original and repeat scans. Per this definition, of the 1,116 ICC values calculated across all extracted radiomic metrics and acquisition settings, 59% were classified as excellent and 13% as good. Nine percent of the values were classified as fair, while 19% were poor (Figure 5). Although high ICC values were pervasive throughout the entire dataset, distinct trends in ICC were observed among different feature families (Figure 5). First‐order and GLCM metrics—particularly the former—consistently showed very good repeatability, with 78% of first‐order and 42% of GLCM features demonstrating good or excellent repeatability across all trials. In contrast, GLDM, GLRLM, GLSZM, and NGTDM families demonstrated very poor repeatability (less than 25%) (Figure 5).

In addition to repeatability, the robustness of features to changes in acquisition settings, as quantified by ICC(3,1), was analyzed via pairwise comparisons of 24 different setting combinations of the variables in Table 1. The same thresholds as used for repeatability were used for robustness—features for which the computed median ICC value was greater than or equal to 0.6 across all 24 settings were considered robust (Table 2).

**TABLE 2:** Robust radiomic features and associated feature families (median ICC ≥ 0.6).

Feature family	Radiomic feature
First Order	Robust mean absolute deviation
	Total energy
	90th percentile
	Energy
	Interquartile range
	Kurtosis
GLCM	IMC2
	MCC
	Cluster tendency
	Correlation
GLDM	Autocorrelation
GLRLM	Difference variance

Abbreviations: GLCM, Gray Level Co‐occurrence Matrix; GLDM, Gray Level Dependence Matrix; GLRLM, Gray Level Run Length Matrix; GLSZM, Gray Level Size Zone Matrix; ICC, intraclass correlation coefficient; IMC2, Informational Measure of Correlation 2; MCC, Maximal Correlation Coefficient; NGTDM, Neighborhood Gray Tone Difference Matrix.

Out of the 93 extracted features, only 12 met the ICC criteria for robustness. These features primarily came from the first‐order and GLCM feature families, demonstrating low sensitivity to the CT acquisition parameters that varied in this study.

### 3.3 Regression analysis

The sensitivity of all radiomic features to acquisition parameters was assessed using regression‐based β coefficients. A heatmap was created to visualize these β coefficients across the 12 different image acquisition settings used to obtain CT scans of the CTTA phantom (Figure 4). Out of the 93 extracted features, 31 demonstrated statistically significant linear associations (*p* ≤ 0.05) with dose across all four scan‐mode/tilt combinations. These radiomic features all show a systematic linear dependence on the acquisition parameters varied in this study. This predictable relationship enables straightforward calibration using linear regression to correct for variability across imaging conditions. These “calibratable” features and their corresponding beta coefficients are comprehensively listed in Appendix A, enabling future calibration to correct for acquisition variations.

### 3.4 Intra‐ and inter‐observer variability

The intra‐observer variability analysis, performed on a single volume segmented twice across all slices by the same operator, yielded a median ICC of 0.97, with a lower quartile of 0.91 and an upper quartile of 0.99. The inter‐observer assessment, performed on the same volume segmented by two different operators, demonstrated a comparable median ICC of 0.97, with a lower quartile of 0.86 and an upper quartile of 0.99.

## 4 DISCUSSION

This study systematically evaluated how scan mode, gantry tilt, and radiation dose affect CT radiomic features in an anthropomorphic liver phantom. Helical scans generally exhibited lower dispersion than axial scans, and introducing a 5° tilt reduced dispersion in axial mode but produced mixed effects in helical mode. Although 34% of features showed good or excellent repeatability, only 13% were robust across all acquisition conditions. Additionally, one‐third of all features demonstrated significant linear relationships with the studied parameters, highlighting both their sensitivity to acquisition variation and their potential for calibration. Together, these findings reinforce the need to account for acquisition‐dependent variability—either through standardized protocols or correction strategies—to improve the reproducibility of CT radiomics, particularly within multicenter studies.

Comparing axial–no tilt and helical–no tilt scans isolates scan‐mode effects, independent of tilt. The higher SD and CV observed in axial scans suggest sharper voxel intensity differences in the image data, whereas the continuous spiral acquisition employed in helical scanning introduces inherent averaging that smooths intensity variation in SD and CV. Interestingly, IQR did not exhibit this same pattern, likely because SD and CV are sensitive to extreme voxel intensities whereas IQR is robust to outliers since it excludes them by definition. Thus, dispersion metrics respond differently depending on whether they capture global variation or only the central portion of the distribution.

Introducing a 5° tilt in axial scans reduced first‐order dispersion, a phenomenon likely attributable to increased partial‐volume averaging and blurring along the tilted axis. This greater degree of averaging effectively reduces the overall image noise, which in turn leads to lower values for dispersion metrics. In helical scans, tilt produced inconsistent effects, likely due to interpolation and volumetric overlap inherent to spiral acquisition. The introduction of tilt during CT reconstruction can alter signal composition and can potentially result in shifts in segmentation boundaries, which could further impact the accuracy of subsequent analyses. Consequently, the observed variability in helical scans with tilt might stem from changes in the signal itself rather than merely a reduction in noise, as seen in axial scans. These sampling differences preclude our ability to directly compare tilted axial and helical VOIs and may explain the absence of a consistent trend in the helical–tilt scenario.

The inverse relationship between dose and noise directly translated into lower values for first‐order dispersion metrics, specifically SD, IQR, and CV. The increased photon count at higher doses provides a more robust signal, thereby minimizing random fluctuations and yielding a clearer image with less intrinsic variability. Even modest dose variation altered first‐order distributions, emphasizing the need for dose consistency when comparing radiomic features across different scans or patient cohorts.

Repeatability and robustness data aligned with patterns reported in the literature: first‐order and many GLCM features demonstrated high ICC values, while higher‐order features exhibited lower stability.^25^, ^26^ First‐order features and many GLCM features primarily capture stable, global intensity properties or normalized texture relationships that are minimally impacted by noise fluctuations or geometric sampling differences.^11^ Furthermore, rank‐based distributions (e.g., robust mean absolute deviation and IQR) and normalized co‐occurrence metrics (e.g., Imc2, MCC, and correlation) emphasize overall structural patterns rather than individual gray‐level values, and are therefore inherently robust to acquisition‐induced perturbations. Therefore, in concordance with prior findings, we recommend prioritizing first‐order and GLCM metrics over other radiomic metrics as a general rule to improve the repeatability and robustness of clinical radiomic modeling, particularly in situations where standardization across institutions or time points is critical. In contrast, higher‐order features (GLRLM, GLSZM, GLDM, and NGTDM) exhibited greater sensitivity to these image acquisition parameters and should be used cautiously or excluded unless repeatability and robustness have been established.

Approximately one‐third of the radiomic features demonstrated significant linear associations with acquisition parameters, indicating sensitivity to protocol variation. This widespread sensitivity highlights a need for rigorous standardization or appropriate correction methods when incorporating radiomic features into multicenter studies, as inconsistencies in acquisition protocols can significantly impact feature values and potentially bias results.^7^ However, these linear relationships also offer the possibility of correcting for these inconsistencies in acquisition protocols. These features are calibratable—their significant linear relationship with the parameters varied in this study allows them to be fit to a linear model in order to correct for said variations. To this end, the regression‐derived β coefficients detailed in Appendix A offer a useful resource. These β coefficients can be utilized to inform and develop specific adjustment strategies, thereby helping to mitigate the influence of image acquisition variations and enhance the reliability and generalizability of radiomic analyses in future research endeavors. Once calibrated, future analyses can focus on these significant metrics, in tandem with the repeatable (Figure 5) and robust (Table 2) metrics identified in this study.

Previous studies have documented the influence of slice thickness, reconstruction algorithm, and tube voltage on radiomic features, often emphasizing dose‐dependent noise effects and scan mode‐specific variability.^4^, ^7^, ^9^ Our study builds on this work by systematically examining the effects of tilt—a relatively less‐studied parameter—demonstrating that even modest tilt angles, such as the 5° tilt introduced in this study, can introduce interpolation artifacts or VOI boundary shifts, particularly in helical scans. This is particularly relevant given that gantry tilt is sometimes necessary in clinical CT acquisitions to minimize radiation to sensitive organs.^27^ In our study, the effect of tilt was simulated at the CT reconstruction stage, rather than at acquisition. However, introducing tilt at any stage can decrease image quality and introduce artifacts that are challenging to correct.^27^ By understanding the acquisition‐dependent sensitivity of various radiomics features, researchers can make informed decisions when selecting metrics of radiomics analysis. This knowledge empowers them to choose more robust and less sensitive features, which, as reported in the literature, helps to reduce the incidence of false positives and significantly improves the overall reproducibility and reliability of radiomic analyses across different institutions and over time.^8^

This study has some limitations, mainly the limited sample size of segmentation operators, the phantom‐only design, and the introduction of tilt during reconstruction. First, although manual segmentation is widely used in radiomic studies, it introduces potential intra‐ and inter‐observer variability.^28^ This variability affects feature stability, particularly shape and texture features, undermining reproducibility and model generalizability.^29^ Semi‐automated and fully automated segmentation tools are promising alternatives that reduce operator bias, speed up workflow, and often improve reproducibility.^30^ However, they are not a panacea, and still require validation and quality control.^30^ Because this work used circular ROIs of fixed size on a phantom with well‐defined boundaries, this variability was expected to be lower than in clinical imaging, where anatomy is more complex. Segmentation variability may be greater in clinical datasets containing heterogeneous pathology. Nevertheless, even in phantom‐only studies, small differences in ROI positioning, slice selection, or partial‐volume boundaries can still influence feature values. Therefore, to add to the rigor of the study, we performed an intra‐ and inter‐observer variability assessment, the results of which indicate excellent reproducibility for both intra‐ and inter‐observer measurements. Second, CTTA phantom‐based results cannot be directly extrapolated to human tissue, which exhibits more complex heterogeneity.^31^ Phantoms provide a controlled environment for isolating acquisition effects by excluding variables present in biological tissue, such as tumor heterogeneity, motion artifacts, tissue deformation, and patient‐dependent noise patterns. Although excluding such variables helps us establish a reproducible benchmark for assessing the technical stability of radiomic features, phantoms lack the biological complexity, anatomical variability, and dynamic contrast present in patient datasets. Therefore, the results of this study cannot be directly extrapolated to biological tissue, and further validation in diverse, multi‐institutional, in vivo cohorts is essential before clinical implementation. Nevertheless, controlled phantom studies are an essential preliminary step before advancing to biological tissue, to ensure that the results are not confounded by potential variables that could be introduced by the use of actual human tissue.^31^ Third, in this study, tilt was introduced during the reconstruction phase via image reslicing.^12^ While reslicing can be implemented on retrospective data and thus allows for convenient exploration of geometric resampling effects on feature extraction, it does not reproduce physical artifacts such as beam hardening or geometric distortions caused by angled x‐ray paths. Therefore, the variability observed in this study may underestimate the full impact of gantry tilt in real‐world CT acquisition. To more accurately replicate the physical effects of gantry tilt in CT imaging, future studies should consider implementing acquisition‐based methods. This can be achieved by using CT scanners with physical gantry tilt capabilities or by leveraging Monte Carlo‐based simulation tools such as CatSim to model realistic beam‐object interactions and X‐ray physics.

Organ motion during CT imaging in a compliant patient is often due to respiration, with the largest movements typically being in the superior–inferior direction.^30^ Average superior to inferior displacement values for the liver vary across studies but are typically in the range of 1–1.5 cm.^32^, ^33^, ^34^ We adopted a 5° tilt during image reconstruction to simulate this characteristic respiration‐induced organ displacement. Future research endeavors could expand upon the current findings by investigating a wider spectrum of tilt angles, which would more accurately simulate the diverse and often subtle variations in patient positioning encountered in real‐world clinical scanning scenarios. This comprehensive analysis could further elucidate the precise impact of image tilt on various radiomic metrics and potentially help identify an acceptable range for tilt, within which radiomic features remain relatively stable and clinically meaningful. It is also important for future studies to consider that the mobility of other organs may differ considerably from the liver, which was the focus of the current simulation.^35^ Organs with greater physiological movement could exhibit enhanced sensitivity to tilt, thus necessitating specific investigations into their unique radiomic responses.^35^ Understanding these organ‐specific dynamics is crucial for developing generalizable correction strategies.

Finally, a critical area for future work involves developing and validating robust correction factors to overcome the data heterogeneity introduced by imaging confounds.^8^ While this study aimed to understand the effects of individual imaging parameters in isolation, real‐world data heterogeneity arises from the complex interplay of multiple acquisition variations.^36^, ^37^ Therefore, exploring comprehensive correction methodologies that can simultaneously account for various imaging confounds will be essential for advancing the clinical utility and reliability of radiomics.^38^

## 5 CONCLUSION

This study highlights the substantial influence of scan mode, tilt, and radiation dose on CT radiomic features, emphasizing the importance of accounting for acquisition variability when designing and interpreting radiomic analyses. Overall, helical mode reduced first‐order noise; tilt introduced complex averaging and interpolation effects; and higher dose improved signal stability. Thirteen out of the 93 radiomic features were identified as being robust. These features primarily belonged to the first‐order and GLCM families, indicating that these feature classes should be prioritized in future studies. The identification of repeatable and robust features, along with 31 calibratable metrics, provides a foundation for parameter correction and harmonization. When interpreting these results, it is important to recognize the inherent limitations in generalizing these findings to in vivo imaging. While this study leveraged a phantom model to evaluate radiomic feature robustness under controlled conditions, phantom studies are merely a foundational—not comprehensive—step toward robust, real‐world radiomics deployment. Future validation in patient datasets is required to confirm that these metrics remain robust when no longer in a controlled phantom setting. Nevertheless, by elucidating the effects of imaging artifacts on radiomic metrics, these findings improve the reliability of multicenter radiomic datasets and promote convergence in the interpretation of radiomic biomarkers across institutions, paving the way for their broader adoption in clinical practice.

## AUTHOR CONTRIBUTIONS

**Neha Yadav**: Primary author of the paper; data processing; and analysis. **Xiaomeng Lei** and **Steven Y. Cen**: Statistical analysis. **Joshua Levy**: Idea and phantom development. **Kristin Jensen**: Imaging protocol development and acquisition of phantom scans. **Bino A. Varghese**: Idea development; analysis; and overall guidance. All authors reviewed the manuscript and approved the final submission.

## CONFLICT OF INTEREST STATEMENT

Joshua Levy is an employee and founder of The Phantom Laboratory, Salem, NY.

## APPENDIX A CALIBRATABLE RADIOMIC FEATURES AND ASSOCIATED REGRESSION COEFFICIENTS (β)

### A.1

These features demonstrated a statistically significant linear association (adjusted *p* ≤ 0.05) with dose across all four scan mode/tilt combinations. These coefficients enable (1) correction of features to a reference acquisition setting, (2) independent evaluation of the directionality and magnitude of acquisition effects on specific features, and (3) application of feature‐level harmonization in other datasets or in multi‐center studies.

**Table:**

		Regression coefficient (β)			
Texture family	Radiomic metric	Axial–no tilt	Axial–tilt	Helical–no tilt	Helical–tilt
First Order	Maximum	−0.160874	−0.212761	−0.218307	−0.105179
	Range	−0.180520	−0.217376	−0.249944	−0.128909
GLCM	Contrast	−0.284723	−0.150132	−0.224372	−0.135103
	Difference average	−0.250416	−0.175968	−0.250777	−0.166521
	Difference entropy	−0.231440	−0.178574	−0.251541	−0.168260
	Difference variance	−0.281444	−0.142941	−0.216816	−0.127351
	Id	0.217558	0.198093	0.272928	0.195522
	Idm	0.223806	0.194155	0.268831	0.189423
	Inverse variance	0.224078	−0.279003	−0.313964	−0.370971
	Joint energy	0.148940	0.193983	0.263009	0.205169
	Joint entropy	−0.204050	−0.180881	−0.250351	−0.167870
	Maximum probability	0.149044	0.199788	0.256728	0.232588
GLRLM	Long run emphasis	0.132629	0.250052	0.308764	0.236799
	Long run low gray level emphasis	0.078193	0.180223	0.280121	0.209680
	Run length non‐uniformity normalized	−0.210095	−0.203956	−0.262213	−0.185337
	Run percentage	−0.199100	−0.222754	−0.281838	−0.208914
	Run variance	0.130963	0.263661	0.320299	0.257479
	Short run emphasis	−0.187329	−0.208899	−0.259867	−0.191819
GLSZM	Gray level non‐uniformity normalized	0.113147	0.188299	0.297331	0.270533
	Gray level variance	−0.210319	−0.191044	−0.207667	−0.192388
	Large area low gray level emphasis	0.068131	0.199195	0.180556	0.230751
	Small area high gray level emphasis	−0.171648	−0.118758	−0.139748	−0.080174
	Zone entropy	−0.119503	−0.209008	−0.297078	−0.222978
	Zone percentage	−0.237409	−0.139616	−0.181794	−0.065719
GLDM	Dependence non‐uniformity normalized	−0.268798	−0.218552	−0.278976	−0.195970
	Dependence variance	0.190314	0.245136	0.324560	0.248039
	Large dependence emphasis	0.176545	0.240586	0.302556	0.235049
	Large dependence low gray level emphasis	0.097469	0.183538	0.283783	0.223601
	Small dependence emphasis	−0.235322	−0.135798	−0.185252	−0.089102
	Small dependence high gray level emphasis	−0.240077	−0.115173	−0.141259	−0.084348
NGTDM	Complexity	−0.216082	−0.157139	−0.200287	−0.090818

Abbreviations: GLCM, Gray Level Co‐occurrence Matrix; GLDM, Gray Level Dependence Matrix; GLRLM, Gray Level Run Length Matrix; GLSZM, Gray Level Size Zone Matrix; NGTDM: Neighborhood Gray Tone Difference Matrix.
